# (*E*)-3-(4-Heptyl­oxyphen­yl)-1-phenyl­prop-2-en-1-one

**DOI:** 10.1107/S1600536813034429

**Published:** 2014-01-18

**Authors:** Davia McKoy, Marion A. Franks, Zerihun Assefa

**Affiliations:** a Department of Chemistry, North Carolina A&T State University, Greensboro, NC 27411, USA

## Abstract

In the title compound, C_22_H_26_O_2_, the aromatic rings are inclined to one another by 8.39 (9)° and the mol­ecule has an *E* conformation about the C=C bond. In the crystal, mol­ecules stack head-to-tail along the *b-*axis direction. They are linked by very weak C—H⋯O contacts, forming *C*(4) chains along [100]. Two chains are linked by a pair of very weak C—H⋯O contacts, enclosing inversion-dimeric *R*_2_^2^(8) ring motifs. There are also C—H⋯π inter­actions present, which link the double-stranded chains, forming a two-dimensional network.

## Related literature   

For general background to chalcones, see: Uchida *et al.* (1998 ▶); Indira *et al.* (2002 ▶); Treadwell (2006 ▶). For their various biological properties, see: Avila *et al.* (2008 ▶); ElSohly *et al.* (2001 ▶); Gafner *et al.* (1996 ▶); Akihisa *et al.* (2003 ▶); Szliszka *et al.* (2009 ▶); Xia *et al.* (2000 ▶); Lahtchev *et al.* (2008 ▶); Bandgar *et al.* (2010 ▶). For their enhanced cytotoxicity towards certain cancers, see: Won *et al.* (2005 ▶). For examples of chalcones with general formula Ar—CH–CH—CO—Ar, with mol­ecular pairing involving π–π inter­actions and hydrogen-bonding, see: Wang *et al.* (2005 ▶). For related halogen derivatives, see: Dutkiewicz *et al.* (2010 ▶); Qiu *et al.* (2006 ▶).
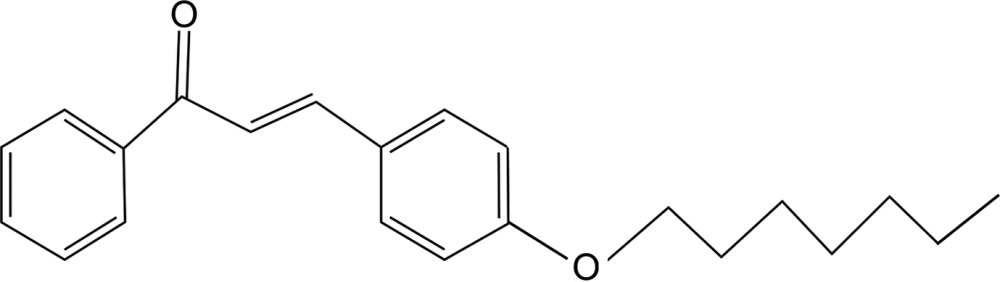


## Experimental   

### 

#### Crystal data  

C_22_H_26_O_2_
*M* *_r_* = 322.43
Triclinic, 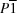

*a* = 5.6069 (9) Å
*b* = 7.7822 (13) Å
*c* = 22.864 (4) Å
α = 81.101 (5)°
β = 85.571 (5)°
γ = 69.879 (4)°
*V* = 925.2 (3) Å^3^
*Z* = 2
Mo *K*α radiation
μ = 0.07 mm^−1^
*T* = 200 K
0.50 × 0.26 × 0.16 mm

#### Data collection  

Bruker X2S diffractometer
Absorption correction: multi-scan (*SADABS*, Bruker, 2005 ▶) *T*_min_ = 0.965, *T*_max_ = 0.989
5754 measured reflections
3149 independent reflections
2314 reflections with *I* > 2σ(*I*)
*R*_int_ = 0.044

#### Refinement  

*R*[*F* ^2^ > 2σ(*F* ^2^)] = 0.051
*wR*(*F* ^2^) = 0.152
*S* = 1.09
3149 reflections
322 parameters
All H-atom parameters refined
Δρ_max_ = 0.44 e Å^−3^
Δρ_min_ = −0.24 e Å^−3^

### 

Data collection: *SMART* (Bruker, 2005 ▶; cell refinement: *SAINT* (Bruker, 2005 ▶); data reduction: *SAINT*; program(s) used to solve structure: *SHELXS97* (Sheldrick, 2008 ▶); program(s) used to refine structure: *SHELXL97* (Sheldrick, 2008 ▶); molecular graphics: *JMol* (Hanson, 2010 ▶); software used to prepare material for publication: *publCIF* (Westrip, 2010 ▶).

## Supplementary Material

Crystal structure: contains datablock(s) I, New_Global_Publ_Block. DOI: 10.1107/S1600536813034429/zp2008sup1.cif

Structure factors: contains datablock(s) I. DOI: 10.1107/S1600536813034429/zp2008Isup2.hkl

Supporting information file. DOI: 10.1107/S1600536813034429/zp2008Isup3.cdx

Supporting information file. DOI: 10.1107/S1600536813034429/zp2008Isup4.cml

CCDC reference:

Additional supporting information:  crystallographic information; 3D view; checkCIF report

## Figures and Tables

**Table 1:** Hydrogen-bond geometry (Å, °) *Cg*1 and *Cg*2 are the centroids of rings C4–C9 and C17–C22, respectively.

*D*—H⋯*A*	*D*—H	H⋯*A*	*D*⋯*A*	*D*—H⋯*A*
C2—H2⋯O1^i^	0.99 (2)	2.67 (2)	3.513 (2)	143.3 (12)
C8—H8⋯O2^ii^	0.95 (2)	2.64 (2)	3.545 (2)	159.3 (13)
C10—H10*B*⋯*Cg*1^iii^	0.98 (2)	2.972 (15)	3.8279 (18)	146.6 (13)
C16—H16*A*⋯*Cg*2^iii^	0.97 (3)	2.97 (2)	3.792 (3)	144.5 (18)

Symmetry codes: (i) 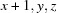; (ii) 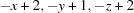; (iii) 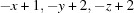.

**Fig. 1.**
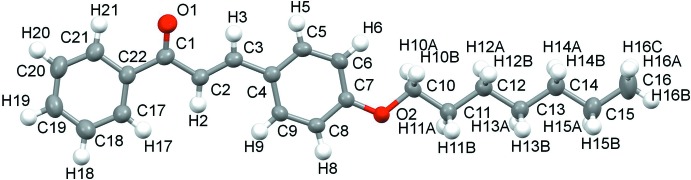
A view of the molecular structure of the title molecule, with atom labelling. Displacement ellipsoids are drawn at the 50% probability level.

## supplementary crystallographic information

### 1. Comment

Chalcones along with their derivatives can easily be obtained by means of isolation from natural products or synthesized by classic scientific methods. These compounds are interesting in the medical field because of their antibacterial (Avila *et al.*, 2008), antifungal (ElSohly *et al.*, 2001; Gafner *et al.*, 1996), antitumor (Akihisa *et al.*, 2003; Szliszka *et al.*, 2009; Xia *et al.*, 2000; Lahtchev *et al.*, 2008) and anti-inflammatory properties (Bandgar *et al.*, 2010). These compounds have also shown enhanced cytotoxicity towards certain cancers (Won *et al.*, 2005). Synthetically chalcones are derived through an aldol condensation which involves the reaction between an aromatic aldehyde with an aliphatic aldehyde or ketone in the presence of a strong base (hydroxide or alkoxide). The resulting compound contains two aromatic rings joined by a three carbon α,β-unsaturated carbonyl system, and we report herein on its crystal structure.

The molecular structure of the title molecule is illustrated in Fig. 1. The two aromatic rings (C4–C9 and C17–C22) are inclined to one another by 8.39 (9)° and the molecule has an *E* conformation about the C2═C3 bond.

In the crystal, the molecules stack head-to-tail along the b axis. They molecules are linked by very weak C-H···O and C-H···π interactions (Table 1). Atom O1 of the carbonyl group interacts with the H atom, H2, of the C2═ C3 double bond in a C═O···HC═C fashion, resulting in the formation of *C*(4) chains along the *a-*axis direction. In addition, the O atom, O2, of the ether moiety is also involved in a weak hydrogen bond with the central phenyl group of an inversion related neighboring molecule. The two molecules are arranged head-to-tail, which induces formation of an inversion dimeric unit and an eight-membered *R*_2_^2^(8) ring containing a pair of very weak C-H···O hydrogen bonds (Table 1).

As a result of the head-to-tail flipping, there is no ring alignment within the structure, hence the system lacks any significant π–π interactions but there are C—H···π contacts present (Table 1) which link the double stranded chains to form a two-dimensional network.

### 2. Experimental

The title compound was obtained by mixing acetophenone (0.150 g, 1.22 mmol), 4-(heptyloxy)benzaldehyde (0.269 g, 1.22 mmol), a 10% solution of NaOH and ethanol at 273 K for 18 h, after which it was acidified with 1 N HCl. The crude product obtained was recrystallized from ethanol yielding yellow plate-like crystals.

### 3. Refinement

All the H atoms were located in difference Fourier maps and freely refined.

### Crystal data

**Table:**

C_22_H_26_O_2_	*Z* = 2
*M**_r_* = 322.43	*F*(000) = 348
Triclinic, *P*1	*D*_x_ = 1.157 Mg m^−^^3^
*a* = 5.6069 (9) Å	Mo *K*α radiation, λ = 0.71073 Å
*b* = 7.7822 (13) Å	Cell parameters from 2314 reflections
*c* = 22.864 (4) Å	θ = 0.9–25.1°
α = 81.101 (5)°	µ = 0.07 mm^−^^1^
β = 85.571 (5)°	*T* = 200 K
γ = 69.879 (4)°	Plate, yellow
*V* = 925.2 (3) Å^3^	0.50 × 0.26 × 0.16 mm

### Data collection

**Table:**

Bruker X2S diffractometer	3149 independent reflections
Radiation source: fine-focus sealed tube	2314 reflections with *I* > 2σ(*I*)
Graphite monochromator	*R*_int_ = 0.044
automatic scans	θ_max_ = 25.1°, θ_min_ = 0.9°
Absorption correction: multi-scan (*SADABS*, Bruker, 2005)	*h* = −6→6
*T*_min_ = 0.965, *T*_max_ = 0.989	*k* = −9→9
5754 measured reflections	*l* = −27→27

### Refinement

**Table:**

Refinement on *F*^2^	Secondary atom site location: difference Fourier map
Least-squares matrix: full	Hydrogen site location: inferred from neighbouring sites
*R*[*F*^2^ > 2σ(*F*^2^)] = 0.051	All H-atom parameters refined
*wR*(*F*^2^) = 0.152	*w* = 1/[σ^2^(*F*_o_^2^) + (0.0807*P*)^2^ + 0.0136*P*] where *P* = (*F*_o_^2^ + 2*F*_c_^2^)/3
*S* = 1.09	(Δ/σ)_max_ = 0.001
3149 reflections	Δρ_max_ = 0.44 e Å^−^^3^
322 parameters	Δρ_min_ = −0.24 e Å^−^^3^
0 restraints	Extinction correction: *SHELXL97* (Sheldrick, 2008), Fc^*^=kFc[1+0.001xFc^2^λ^3^/sin(2θ)]^-1/4^
Primary atom site location: structure-invariant direct methods	Extinction coefficient: 0.055 (8)

### Special details

**Table:**

Geometry. All e.s.d.'s (except the e.s.d. in the dihedral angle between two l.s. planes) are estimated using the full covariance matrix. The cell e.s.d.'s are taken into account individually in the estimation of e.s.d.'s in distances, angles and torsion angles; correlations between e.s.d.'s in cell parameters are only used when they are defined by crystal symmetry. An approximate (isotropic) treatment of cell e.s.d.'s is used for estimating e.s.d.'s involving l.s. planes.
Refinement. Refinement of *F*^2^ against ALL reflections. The weighted *R*-factor *wR* and goodness of fit *S* are based on *F*^2^, conventional *R*-factors *R* are based on *F*, with *F* set to zero for negative *F*^2^. The threshold expression of *F*^2^ > σ(*F*^2^) is used only for calculating *R*-factors(gt) *etc*. and is not relevant to the choice of reflections for refinement. *R*-factors based on *F*^2^ are statistically about twice as large as those based on *F*, and *R*- factors based on ALL data will be even larger.

### Fractional atomic coordinates and isotropic or equivalent isotropic displacement parameters (Å^2^)

**Table:**

	*x*	*y*	*z*	*U*_iso_*/*U*_eq_	
C1	0.0523 (3)	0.7906 (2)	0.72962 (7)	0.0430 (4)	
C2	0.2373 (3)	0.7615 (2)	0.77569 (7)	0.0400 (4)	
C3	0.1563 (3)	0.7911 (2)	0.83070 (7)	0.0387 (4)	
C4	0.3023 (3)	0.7649 (2)	0.88344 (7)	0.0364 (4)	
C5	0.1749 (3)	0.8252 (2)	0.93499 (7)	0.0413 (4)	
C6	0.2981 (3)	0.8036 (2)	0.98702 (8)	0.0416 (4)	
C7	0.5591 (3)	0.7171 (2)	0.98833 (7)	0.0351 (4)	
C8	0.6904 (3)	0.6503 (2)	0.93778 (7)	0.0390 (4)	
C9	0.5660 (3)	0.6753 (2)	0.88623 (7)	0.0382 (4)	
C10	0.5762 (3)	0.7608 (2)	1.08943 (7)	0.0402 (4)	
C11	0.7707 (3)	0.7139 (2)	1.13640 (7)	0.0417 (4)	
C12	0.6526 (3)	0.7708 (2)	1.19529 (7)	0.0434 (5)	
C13	0.8447 (4)	0.7246 (2)	1.24366 (8)	0.0449 (5)	
C14	0.7285 (4)	0.7721 (2)	1.30362 (8)	0.0471 (5)	
C15	0.9149 (4)	0.7081 (3)	1.35305 (9)	0.0608 (6)	
C16	0.7959 (7)	0.7512 (4)	1.41297 (10)	0.0790 (7)	
C17	0.3892 (4)	0.6901 (3)	0.64939 (8)	0.0485 (5)	
C18	0.4548 (4)	0.6707 (3)	0.59057 (9)	0.0582 (5)	
C19	0.2705 (4)	0.7373 (3)	0.54822 (9)	0.0596 (6)	
C20	0.0207 (4)	0.8227 (3)	0.56481 (8)	0.0580 (6)	
C21	−0.0447 (4)	0.8401 (3)	0.62334 (8)	0.0492 (5)	
C22	0.1388 (3)	0.7733 (2)	0.66679 (7)	0.0403 (4)	
O1	−0.1746 (2)	0.8262 (2)	0.74253 (5)	0.0659 (5)	
O2	0.7021 (2)	0.68945 (15)	1.03686 (5)	0.0435 (3)	
H2	0.421 (4)	0.718 (2)	0.7647 (7)	0.049 (5)*	
H3	−0.021 (4)	0.828 (2)	0.8379 (7)	0.047 (5)*	
H5	−0.003 (3)	0.881 (2)	0.9330 (7)	0.044 (5)*	
H6	0.206 (3)	0.843 (2)	1.0227 (8)	0.051 (5)*	
H8	0.867 (3)	0.587 (2)	0.9416 (7)	0.040 (4)*	
H9	0.660 (3)	0.629 (2)	0.8518 (7)	0.043 (4)*	
H10A	0.444 (3)	0.700 (2)	1.1034 (7)	0.053 (5)*	
H10B	0.496 (3)	0.895 (2)	1.0810 (7)	0.044 (4)*	
H11A	0.898 (3)	0.775 (2)	1.1222 (7)	0.049 (5)*	
H11B	0.860 (3)	0.578 (2)	1.1418 (7)	0.042 (4)*	
H12A	0.534 (4)	0.700 (2)	1.2111 (8)	0.059 (5)*	
H12B	0.563 (3)	0.899 (3)	1.1912 (8)	0.052 (5)*	
H13A	0.967 (4)	0.790 (2)	1.2316 (8)	0.058 (5)*	
H13B	0.938 (4)	0.588 (3)	1.2473 (8)	0.059 (5)*	
H14A	0.644 (3)	0.901 (3)	1.3017 (7)	0.051 (5)*	
H14B	0.601 (4)	0.711 (3)	1.3149 (8)	0.064 (6)*	
H15A	1.048 (4)	0.763 (3)	1.3422 (8)	0.070 (6)*	
H15B	0.994 (4)	0.571 (3)	1.3551 (9)	0.076 (6)*	
H16A	0.719 (4)	0.880 (3)	1.4161 (9)	0.078 (7)*	
H16B	0.917 (5)	0.698 (4)	1.4446 (13)	0.119 (10)*	
H16C	0.656 (5)	0.692 (4)	1.4224 (12)	0.121 (10)*	
H17	0.517 (3)	0.644 (2)	0.6772 (8)	0.048 (5)*	
H18	0.630 (4)	0.611 (2)	0.5772 (8)	0.064 (6)*	
H19	0.319 (4)	0.725 (3)	0.5065 (9)	0.068 (6)*	
H20	−0.115 (4)	0.867 (3)	0.5363 (9)	0.067 (6)*	
H21	−0.219 (4)	0.898 (3)	0.6362 (8)	0.060 (6)*	

### Atomic displacement parameters (Å^2^)

**Table:**

	*U*^11^	*U*^22^	*U*^33^	*U*^12^	*U*^13^	*U*^23^
C1	0.0374 (11)	0.0448 (9)	0.0462 (10)	−0.0142 (8)	−0.0048 (8)	−0.0014 (7)
C2	0.0357 (11)	0.0423 (9)	0.0413 (10)	−0.0133 (8)	−0.0032 (8)	−0.0020 (7)
C3	0.0334 (10)	0.0376 (8)	0.0438 (10)	−0.0111 (7)	−0.0014 (8)	−0.0036 (7)
C4	0.0374 (10)	0.0317 (8)	0.0400 (9)	−0.0119 (7)	−0.0008 (7)	−0.0044 (7)
C5	0.0318 (10)	0.0410 (9)	0.0486 (11)	−0.0074 (8)	0.0003 (8)	−0.0111 (7)
C6	0.0393 (11)	0.0432 (9)	0.0406 (10)	−0.0095 (8)	0.0040 (8)	−0.0135 (7)
C7	0.0373 (10)	0.0312 (7)	0.0350 (9)	−0.0095 (7)	−0.0003 (7)	−0.0041 (6)
C8	0.0325 (10)	0.0401 (9)	0.0399 (9)	−0.0072 (8)	0.0010 (8)	−0.0046 (7)
C9	0.0366 (10)	0.0397 (8)	0.0351 (9)	−0.0094 (7)	0.0032 (8)	−0.0057 (7)
C10	0.0432 (11)	0.0379 (9)	0.0368 (9)	−0.0094 (8)	0.0036 (8)	−0.0091 (7)
C11	0.0459 (11)	0.0386 (9)	0.0380 (10)	−0.0109 (8)	−0.0013 (8)	−0.0052 (7)
C12	0.0455 (11)	0.0410 (9)	0.0403 (10)	−0.0096 (8)	−0.0023 (8)	−0.0066 (7)
C13	0.0493 (11)	0.0390 (9)	0.0425 (10)	−0.0088 (8)	−0.0060 (8)	−0.0059 (7)
C14	0.0545 (12)	0.0414 (10)	0.0426 (10)	−0.0120 (9)	−0.0046 (9)	−0.0055 (8)
C15	0.0751 (15)	0.0541 (12)	0.0495 (12)	−0.0130 (11)	−0.0172 (11)	−0.0088 (9)
C16	0.119 (2)	0.0727 (16)	0.0472 (13)	−0.0301 (16)	−0.0154 (14)	−0.0122 (11)
C17	0.0422 (12)	0.0569 (10)	0.0458 (11)	−0.0149 (9)	−0.0054 (9)	−0.0073 (8)
C18	0.0522 (13)	0.0721 (13)	0.0516 (12)	−0.0205 (10)	0.0036 (10)	−0.0159 (10)
C19	0.0697 (15)	0.0743 (13)	0.0412 (11)	−0.0317 (12)	0.0002 (10)	−0.0105 (10)
C20	0.0625 (15)	0.0695 (13)	0.0449 (12)	−0.0264 (11)	−0.0144 (10)	−0.0007 (9)
C21	0.0446 (12)	0.0557 (11)	0.0475 (11)	−0.0181 (9)	−0.0078 (9)	−0.0022 (8)
C22	0.0438 (11)	0.0398 (9)	0.0404 (9)	−0.0186 (8)	−0.0052 (8)	−0.0028 (7)
O1	0.0394 (9)	0.1036 (11)	0.0515 (8)	−0.0195 (7)	−0.0026 (6)	−0.0110 (7)
O2	0.0394 (7)	0.0494 (7)	0.0347 (7)	−0.0047 (5)	−0.0026 (5)	−0.0088 (5)

### Geometric parameters (Å, º)

**Table:**

C1—O1	1.228 (2)	C12—H12A	1.014 (18)
C1—C2	1.473 (2)	C12—H12B	0.940 (18)
C1—C22	1.491 (2)	C13—C14	1.515 (2)
C2—C3	1.331 (2)	C13—H13A	0.986 (18)
C2—H2	0.991 (19)	C13—H13B	1.005 (19)
C3—C4	1.459 (2)	C14—C15	1.506 (3)
C3—H3	0.944 (18)	C14—H14A	0.947 (19)
C4—C5	1.389 (2)	C14—H14B	0.99 (2)
C4—C9	1.403 (2)	C15—C16	1.510 (3)
C5—C6	1.380 (2)	C15—H15A	0.98 (2)
C5—H5	0.944 (18)	C15—H15B	1.00 (2)
C6—C7	1.385 (2)	C16—H16A	0.95 (2)
C6—H6	0.963 (18)	C16—H16B	0.97 (3)
C7—O2	1.3659 (19)	C16—H16C	1.03 (3)
C7—C8	1.393 (2)	C17—C18	1.384 (3)
C8—C9	1.370 (2)	C17—C22	1.385 (3)
C8—H8	0.945 (18)	C17—H17	0.934 (18)
C9—H9	0.959 (17)	C18—C19	1.380 (3)
C10—O2	1.4343 (19)	C18—H18	0.98 (2)
C10—C11	1.504 (2)	C19—C20	1.379 (3)
C10—H10A	1.019 (17)	C19—H19	0.982 (19)
C10—H10B	0.977 (17)	C20—C21	1.375 (3)
C11—C12	1.514 (2)	C20—H20	0.97 (2)
C11—H11A	0.992 (17)	C21—C22	1.395 (2)
C11—H11B	0.995 (17)	C21—H21	0.966 (19)
C12—C13	1.519 (2)		
			
O1—C1—C2	120.52 (15)	H12A—C12—H12B	110.1 (16)
O1—C1—C22	119.01 (15)	C14—C13—C12	114.23 (15)
C2—C1—C22	120.45 (15)	C14—C13—H13A	108.9 (10)
C3—C2—C1	119.86 (16)	C12—C13—H13A	109.1 (11)
C3—C2—H2	121.6 (10)	C14—C13—H13B	108.6 (10)
C1—C2—H2	118.5 (10)	C12—C13—H13B	107.0 (10)
C2—C3—C4	129.51 (17)	H13A—C13—H13B	108.9 (15)
C2—C3—H3	116.6 (10)	C15—C14—C13	114.37 (17)
C4—C3—H3	113.8 (10)	C15—C14—H14A	109.2 (10)
C5—C4—C9	117.15 (15)	C13—C14—H14A	110.3 (10)
C5—C4—C3	118.98 (15)	C15—C14—H14B	106.2 (11)
C9—C4—C3	123.82 (15)	C13—C14—H14B	108.9 (10)
C6—C5—C4	122.69 (17)	H14A—C14—H14B	107.6 (16)
C6—C5—H5	120.8 (10)	C14—C15—C16	114.0 (2)
C4—C5—H5	116.5 (10)	C14—C15—H15A	107.9 (12)
C5—C6—C7	118.98 (16)	C16—C15—H15A	111.3 (12)
C5—C6—H6	121.5 (11)	C14—C15—H15B	107.1 (12)
C7—C6—H6	119.5 (11)	C16—C15—H15B	108.3 (11)
O2—C7—C6	124.38 (14)	H15A—C15—H15B	108.1 (18)
O2—C7—C8	116.11 (14)	C15—C16—H16A	114.9 (13)
C6—C7—C8	119.51 (15)	C15—C16—H16B	112.3 (17)
C9—C8—C7	120.79 (16)	H16A—C16—H16B	107 (2)
C9—C8—H8	123.0 (9)	C15—C16—H16C	108.7 (15)
C7—C8—H8	116.2 (9)	H16A—C16—H16C	107 (2)
C8—C9—C4	120.84 (16)	H16B—C16—H16C	106 (2)
C8—C9—H9	119.5 (10)	C18—C17—C22	120.81 (18)
C4—C9—H9	119.7 (10)	C18—C17—H17	118.4 (11)
O2—C10—C11	108.43 (13)	C22—C17—H17	120.8 (11)
O2—C10—H10A	108.8 (9)	C19—C18—C17	120.1 (2)
C11—C10—H10A	109.4 (10)	C19—C18—H18	117.5 (11)
O2—C10—H10B	109.6 (9)	C17—C18—H18	122.4 (11)
C11—C10—H10B	109.8 (10)	C20—C19—C18	119.71 (19)
H10A—C10—H10B	110.8 (14)	C20—C19—H19	120.7 (12)
C10—C11—C12	112.39 (14)	C18—C19—H19	119.6 (12)
C10—C11—H11A	108.4 (10)	C21—C20—C19	120.16 (19)
C12—C11—H11A	110.8 (9)	C21—C20—H20	118.0 (12)
C10—C11—H11B	108.5 (9)	C19—C20—H20	121.8 (12)
C12—C11—H11B	108.7 (9)	C20—C21—C22	120.95 (19)
H11A—C11—H11B	107.9 (14)	C20—C21—H21	121.8 (11)
C11—C12—C13	113.55 (15)	C22—C21—H21	117.3 (11)
C11—C12—H12A	110.2 (10)	C17—C22—C21	118.26 (16)
C13—C12—H12A	104.3 (10)	C17—C22—C1	123.77 (15)
C11—C12—H12B	110.4 (11)	C21—C22—C1	117.95 (15)
C13—C12—H12B	108.2 (11)	C7—O2—C10	118.10 (12)

### Hydrogen-bond geometry (Å, º)

Cg1 and Cg2 are the centroids of rings C4–C9 and C17–C22, respectively.

**Table:**

*D*—H···*A*	*D*—H	H···*A*	*D*···*A*	*D*—H···*A*
C2—H2···O1^i^	0.99 (2)	2.67 (2)	3.513 (2)	143.3 (12)
C8—H8···O2^ii^	0.95 (2)	2.64 (2)	3.545 (2)	159.3 (13)
C10—H10*B*···*Cg*1^iii^	0.98 (2)	2.972 (15)	3.8279 (18)	146.6 (13)
C16—H16*A*···*Cg*2^iii^	0.97 (3)	2.97 (2)	3.792 (3)	144.5 (18)

Symmetry codes: (i) *x*+1, *y*, *z*; (ii) −*x*+2, −*y*+1, −*z*+2; (iii) −*x*+1, −*y*+2, −*z*+2.
